# Scars of COVID-19: A bibliometric analysis of post-COVID-19 fibrosis

**DOI:** 10.3389/fpubh.2022.967829

**Published:** 2022-09-20

**Authors:** Han Zhong, Yang Zhou, Shu-Ya Mei, Ri Tang, Jin-Hua Feng, Zheng-Yu He, Qiao-Yi Xu, Shun-Peng Xing

**Affiliations:** Department of Critical Care Medicine, Renji Hospital, School of Medicine, Shanghai Jiao Tong University, Shanghai, China

**Keywords:** COVID-19, fibrosis, etiology, therapy, hotspots and trends

## Abstract

**Background:**

The coronavirus disease 2019 (COVID-19) becomes a worldwide public health threat. Increasing evidence proves that COVID-19-induced acute injuries could be reversed by a couple of therapies. After that, post-COVID-19 fibrosis (PCF), a sequela of “Long COVID,” earns rapidly emerging concerns. PCF is associated with deteriorative lung function and worse quality of life. But the process of PCF remains speculative. Therefore, we aim to conduct a bibliometric analysis to explore the overall structure, hotspots, and trend topics of PCF.

**Materials and methods:**

A comprehensive search was performed in the Web of Science core database to collect literature on PCF. Search syntax included COVID-19 relevant terms: “COVID 19,” “COVID-19 Virus Disease,” “COVID-19 Virus Infection,” “Coronavirus Disease-19,” “2019 Novel Coronavirus Disease,” “2019 Novel Coronavirus Infection,” “SARS Coronavirus 2 Infection,” “COVID-19 Pandemic,” “Coronavirus,” “2019-nCoV,” and “SARS-CoV-2”; and fibrosis relevant terms: “Fibrosis,” “Fibroses,” and “Cirrhosis.” Articles in English were included. Totally 1,088 publications were enrolled. Searching results were subsequentially exported and collected for the bibliometric analysis. National, organizational, and individual level data were analyzed and visualized through biblioshiny package in the R, VOSviewer software, the CiteSpace software, and the Graphical Clustering Toolkit (gCLUTO) software, respectively.

**Results:**

The intrinsic structure and development in the field of PCF were investigated in the present bibliometric analysis. The topmost keywords were “COVID-19” (occurrences, 636) surrounded by “SARS-CoV-2” (occurrences, 242), “coronavirus” (occurrences, 123), “fibrosis” (occurrences, 120), and “pneumonia” (occurrences, 94). The epidemiology, physiopathology, diagnosis, and therapy of PCF were extensively studied. After this, based on dynamic analysis of keywords, hot topics sharply changed from “Wuhan,” “inflammation,” and “cytokine storm” to “quality of life” and “infection” through burst detection; from “acute respiratory syndrome,” “cystic-fibrosis” and “fibrosis” to “infection,” “COVID-19,” “quality-of-life” through thematic evolution; from “enzyme” to “post COVID.” Similarly, co-cited references analysis showed that topics of references with most citations shift from “pulmonary pathology” (cluster 0) to “COVID-19 vaccination” (cluster 6). Additionally, the overview of contributors, impact, and collaboration was revealed. Summarily, the USA stood out as the most prolific, influential, and collaborative country. The Udice French Research University, Imperial College London, Harvard University, and the University of Washington represented the largest volume of publications, citations, *H*-index, and co-authorships, respectively. Dana Albon was the most productive and cited author with the strongest co-authorship link strength. *Journal of Cystic Fibrosis* topped the list of prolific and influential journals.

**Conclusion:**

Outcomes gained from this study assisted professionals in better realizing PCF and would guide future practices. Epidemiology, pathogenesis, and therapeutics were study hotspots in the early phase of PCF research. As the spread of the COVID-19 pandemic and progress in this field, recent attention shifted to the quality of life of patients and post-COVID comorbidities. Nevertheless, COVID-19 relevant infection and vaccination were speculated to be research trends with current and future interest. International cooperation as well as in-depth laboratory experiments were encouraged to promote further explorations in the field of PCF.

## Introduction

According to data from the World Health Organization, as of 29 May 2022, the coronavirus disease 2019 (COVID-19) spread globally with over 526 million confirmed cases and over six million deaths (1). The pandemic is caused by severe acute respiratory syndrome coronavirus 2 (SARS-CoV-2), which results in symptoms vary from asymptomatic infection to multiple organ dysfunction syndrome (2). As COVID-19 evolves, emerging evidence has demonstrated that the acute phase could be reversed by prolonged low-dose corticosteroids, anticoagulation, and proactive oxygen supports (3). Particularly, corticosteroids including dexamethasone, hydrocortisone, methylprednisolone, and prednisone are found to reduce mortality in patients with severe and critical COVID-19 (4). Therefore, the majority of patients are expected to recover from SARS-CoV-2 infections (5). It has been increasingly important to investigate the long-term consequences of COVID-19 (6).

Fibrosis is a recognized sequela of “Long COVID” (6–8). Early data suggest a high rate (25–47%) of fibrotic abnormalities in COVID-19 patients (9–11). However, the mechanism underlying PCF remains speculative. Acute respiratory distress syndrome (ARDS) secondary to COVID-19 is reported as the largest contributor to PCF (12). In the course of ARDS, pulmonary fibrosis occurred as early as <1 week (13). The pathogenesis underlying is complex (6). Prolonged mechanical ventilation (MV) (14, 15), epithelial injury (16), and endothelial injury (17) may activate pro-fibrotic responses. Cytokine storm is considered an essential element in the PCF process (18, 19). Excessive release of cytokine tumor necrosis factor-α (TNF-α) initiates fibrosis and lung remodeling (20). Also, the SARS-CoV-2 virus is documented to directly trigger fibrosis through epidermal growth factor receptor signaling (6, 21).

PCF is associated with deteriorative lung function and worse quality of life (11, 22). Thus, PCF needs early recognition and a holistic package of care (23–25). Although a couple of studies report functional and radiologic changes in post-COVID-19 fibrotic patients (11, 26, 27), a comprehensive overview and dynamic analysis of these literatures are absent (28). Currently, we attempt to investigate the most influential contributors and articles in this research field, analysis of the characteristics of topmost keywords on PCF and identify relevant burstiness, and provide a definitive insight into the research in the field of PCF using the bibliometric methodology. This analysis will survey the historical footprints and overall structure of the research on PCF, highlight hotspots and potential future trends, and guide researchers in conducting further practices in the field of PCF.

## Materials and methods

### Data collection

A comprehensive search was performed in the Web of Science (WOS) core database on 12 July 2022. The search syntax was consisted of COVID-19 relevant terms: “COVID 19,” “COVID-19 Virus Disease,” “COVID-19 Virus Infection,” “Coronavirus Disease-19,” “2019 Novel Coronavirus Disease,” “2019 Novel Coronavirus Infection,” “SARS Coronavirus 2 Infection,” “COVID-19 Pandemic,” “Coronavirus,” “2019-nCoV,” and “SARS-CoV-2”; and fibrosis relevant terms: “Fibrosis,” “Fibroses,” and “Cirrhosis.” Articles in English were included. Searching results were exported *via* both plain text and Microsoft Excel files. In this research, various tags such as title, author, source, abstract, and citation record were collected for the bibliometric analysis.

### Bibliometric analysis

Bibliometric analysis is a mathematical and statistical research method to quantify scientific production and impact (29). Thanks to this analysis, we are able to analyze and visualize different levels of co-authorship, keyword co-occurrence, thematic evolution, document co-citation, category assignment, citations, and other bibliographic parameters (30). Accordingly, bibliometric analyses highlight the landscape of historical research, shape existing practice in a specific field, and provide recommendations for future research work (31, 32).

This study used scientific bibliometric tools including VOSviewer (version 1.6.10, Leiden University, Netherlands), biblioshiny package in R (version 4.2.1) (33), Graphical Clustering Toolkit (gCLUTO) software (Version 1.0) (University of Minnesota, USA), and CiteSpace software (version 6.1.R2) (34).

VOSviewer is a widely used analytical and graphical software. Co-occurrence analysis can find out high-density keywords and speculate hot topics of research. This co-occurrence analysis was performed using a text file retrieved from the WOS core database. For clarity, keywords were included with a threshold of more than 10 frequencies. Multiple appearances of keywords were recognized as a single one and uniformized by enrolling thesaurus terms. The network and clusters of keywords were subsequently generated. In the network map, the color of bubbles indicated a group of keywords, bubble size represented the volume of article counts, and distance between two bubbles indicated the frequency of co-occurrence.

Co-authorship analysis of scientific articles screened connections between countries/regions, organizations, and authors. This analytical approach could contribute to assess partnerships and collaborations between producers at different levels. Similarly, the bubble size and color represented publication count and cluster, respectively. The thickness of the line between two bubbles revealed the linkage strength of contributors.

The overlay visualization map of theme words was generated through VOSviewer based on title and abstract words. The words that appeared more than 50 times were enrolled and binary counted. The bubble colors indicated the average publication date of particular words. The purple color indicated the former publication date. The yellow color indicated a later publication date. Therefore, the map showed the shift trends of highly frequent theme words.

The CiteSpace software, a robust mathematical analysis, and a machine learning tool were employed to conduct keywords burst detection. Burst detection is a function detecting whether and when the burstiness occurred. The burstiness was evaluated by sum appearance weighted by the time window. The surging frequency of a keyword indicated sharply increased attention to a particular topic (30, 35).

Reference co-citation time view was generated by the CiteSpace software as well. It was based on the hypothesis that co-cited reference revealed similar study trends (34). In the timeline maps, some indicators were used. The weighted mean silhouette is a metric graphically elucidating cluster. The score of silhouette ranges from −1 to 1, the higher value means larger inter-cluster consistency and homogeneity (35). Modularity was employed to assess the community of clusters. The value ranges between 0 and 1. The higher value means better clustering, while lower modularity indicates worse communities (30).

Biblioshiny is an augmented tool kit in R for mathematical calculation and visualization. In this work, biblioshiny was employed to generate a thematic evolution map. Through coword analysis, keywords in distinct articles are displayed in a low-dimensional space according to the different time span of each article. Therefore, the temporary changes of keywords accumulated topic changes, and the thematic evolution map revealed the trends of research development (36).

Production and impact of contributors were assessed using the intrinsic analysis tool in WOS and Biblioshiny, respectively. The volume of publications, citations, and *H*-index of countries/regions and institutions were retrieved from WOS. The *H*-index of authors and journals was calculated through the impact analysis tool in Biblioshiny. The H-index is a well-known indicator of productivity. It is widely used to compare the contribution and impact of producers in similar research fields (29).

In addition, bi-clustering was performed using the gCLUTO software. Highly frequent keywords/source documents matrix were graphically clustered and interpreted the internal semantic relationships and emerging study areas. The parameters were set as follows: Clustering method: Repeated bisection; Criterion function: I2; Similarity function: Cosine; Graph Model: Asymmetric-Direct. The clustering was repeated to optimize the matrix with acceptable values of Isim and Esim (37).

In addition, the method regarding overlay visualization of thematic words (VOSviewer), thematic map, trend topics, country collaboration map, and author/journal impact ranking map (biblioshiny) were described in the Supplementary Figures 1–4.

## Results

### Topmost keywords

**Figure 1:**
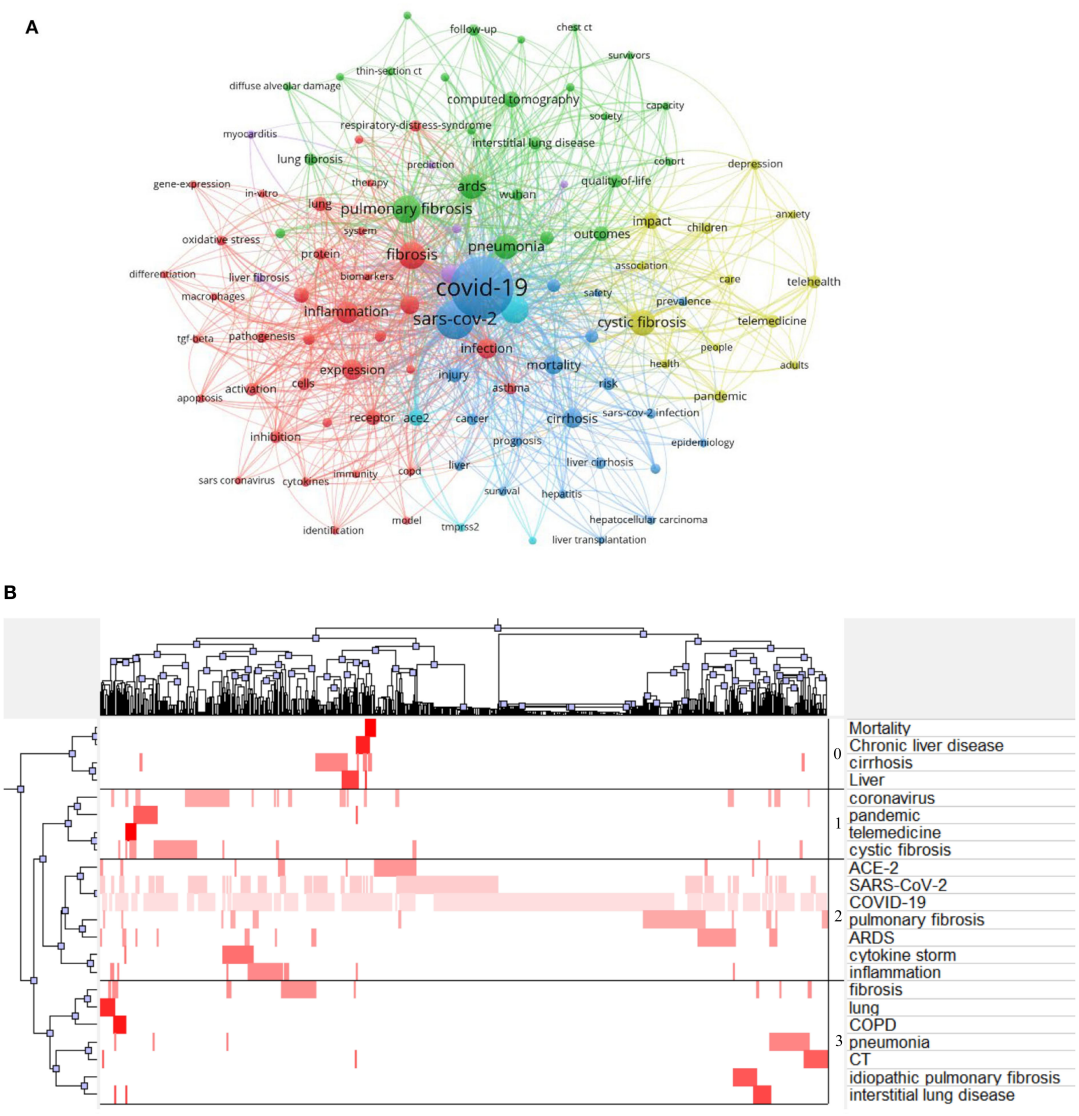
Topmost keywords. **(A)** A network of most frequent keywords was generated by VOSviewer, different colors classified clusters, bubble size indicated publication amount, and thickness of the line revealed linkage strength between keywords. **(B)** Bi-clustering matrix was generated by Graphical Clustering Toolkit, *X*-axis indicated the sequence number of publications, and *Y*-axis represented high-frequency keywords. The tree indicated connections between publications or high-frequency keywords. The darker color of the red blocks revealed a higher appearance of high-frequency keywords in a particular article.

A total of 3,791 keywords were identified from 1,093 enrolled articles. The VOSviewer was used to generate a network map. Keywords with more than 10 appearances were defined as highly frequent keywords. There were 105 most frequent keywords divisible into six clusters. As shown in Figure 1A, “COVID-19” (occurrences, 636; link strength, 1901) was surrounded by “SARS-CoV-2” (occurrences, 242; link strength, 861), “coronavirus” (occurrences, 123; link strength, 475), “fibrosis” (occurrences, 120; link strength, 460), “pneumonia” (occurrences, 94; link strength, 349), “infection” (occurrences, 64; link strength, 251), “diagnosis” (occurrences, 27; link strength, 115), and “dysfunction” (occurrences, 15; link strength, 58). The ranking of the top 10 keywords with the largest occurrence is shown in Table 1.

**Table 1:** The ranking of the top 10 keywords with largest occurrences in the field of COVID-19-associated fibrosis.

**Rank**	**Keyword**	**Occurrences**	**Total link strength**
1	COVID-19	636	1901
2	SARS-CoV-2	242	861
3	Pulmonary fibrosis	124	398
4	Coronavirus	123	475
5	Fibrosis	120	460
6	Cystic fibrosis	110	310
7	ARDS	102	438
8	Pneumonia	94	349
9	Inflammation	80	335
10	Expression	68	302

Bi-clustering analysis was conducted to identify research hotspots in this area. There were 22 highly frequent terms with more than ten appearances. A two-dimensional matrix was generated indicating the highly frequent keyword and relevant source article. Subsequently, the matrix was bi-clustered into four highly divisible clusters using the gCLUTO software. Topics of each cluster were artificially analyzed by screening the clustered keywords (Figure 1B) and denominated as follows:

Cluster 0: Epidemiology of fibrosis due to COVID-19.
Cluster 1: Therapy for post-COVID-19 fibrosis.
Cluster 2: The etiology and physiopathology of pulmonary fibrosis after COVID-19 illness.
Cluster 3: Diagnosis of fibrotic lung disease complicated with COVID-19.

Similarly, a two-dimensional thematic map was generated interpreting the activity and importance of theme words (Supplementary Figure 1). A cluster including “expression,” “inflammation,” “receptor,” “pulmonary-fibrosis,” and “activation” was considered well-developed and important.

### Dynamic keywords and thematic evolution

**Figure 2:**
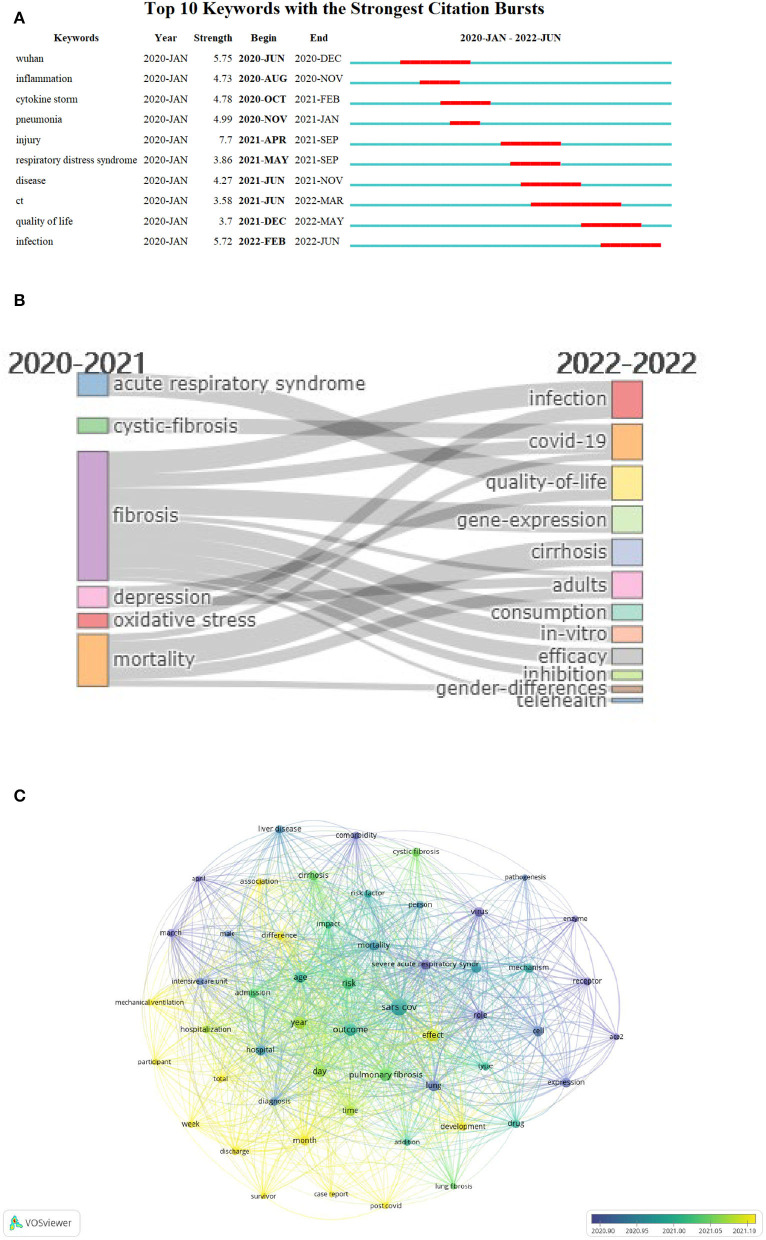
Time view of keywords. **(A)** Top 10 most bursting keywords between 2020 and 2022. The red bar indicated the appearance time span of keywords. **(B)** Thematic evolution between 2020 and 2022. The cutting point of time slices is 2021. **(C)** Overlay visualization of thematic terms. The bubble colors indicated the average publication date of particular words. The purple color indicated the former publication date. The yellow color indicated later publication date.

Burst detection of keywords was conducted to show the dynamic trends of the most bursting keywords. In the time view, there were10 keywords with the strongest citation burst ranking in ascending order of start month. Keyword “ct” (abbreviation of “computerized tomography”) presented the longest burst from June 2021 to March 2022, which was important in identifying PCF. In the early phase of PCF research, “Wuhan,” “inflammation,” “cytokine storm,” and “pneumonia” observed the strongest bursts. In the developing period, the keywords “injury,” “respiratory distress syndrome,” “disease,” and “ct” earned the most attention. Recently, “quality of life” received emerging interest, while “infection” represented recent and future research trends (Figure 2A).

The thematic evolution map revealed that research topics of PCF altered largely from 2020 to 2022. Before 2021, “acute respiratory syndrome,” “cystic-fibrosis” “fibrosis,” “depression,” “oxidative stress,” and “mortality” were the hotspots in the field of PCF. In 2022, the hotspots changed to “infection,” “COVID-19,” “quality-of-life,” “gene-expression,” “cirrhosis,” “adults,” “consumption,” “in-vitro,” “efficacy,” “inhibition,” “gender-differences,” and “telehealth” (Figure 2B).

Overlay visualization map of theme words represented that “enzyme” (average publication year: 2020.80), “ace2” (abbreviation of angiotensin-converting enzyme 2; average publication year: 2020.81), “virus” (average publication year: 2020.86) earned notable attention in the early phase of PCF research. Lately, the study emphasis has shifted to “post COVID” (average publication year: 2021.32), “month” (average publication year: 2021.30), and “total” (average publication year: 2021.20) (Figure 2C).

In addition, the trend topic map presented a similar change from “pneumonia” to “society” (Supplementary Figure 2).

### Analysis of co-cited references

**Figure 3:**
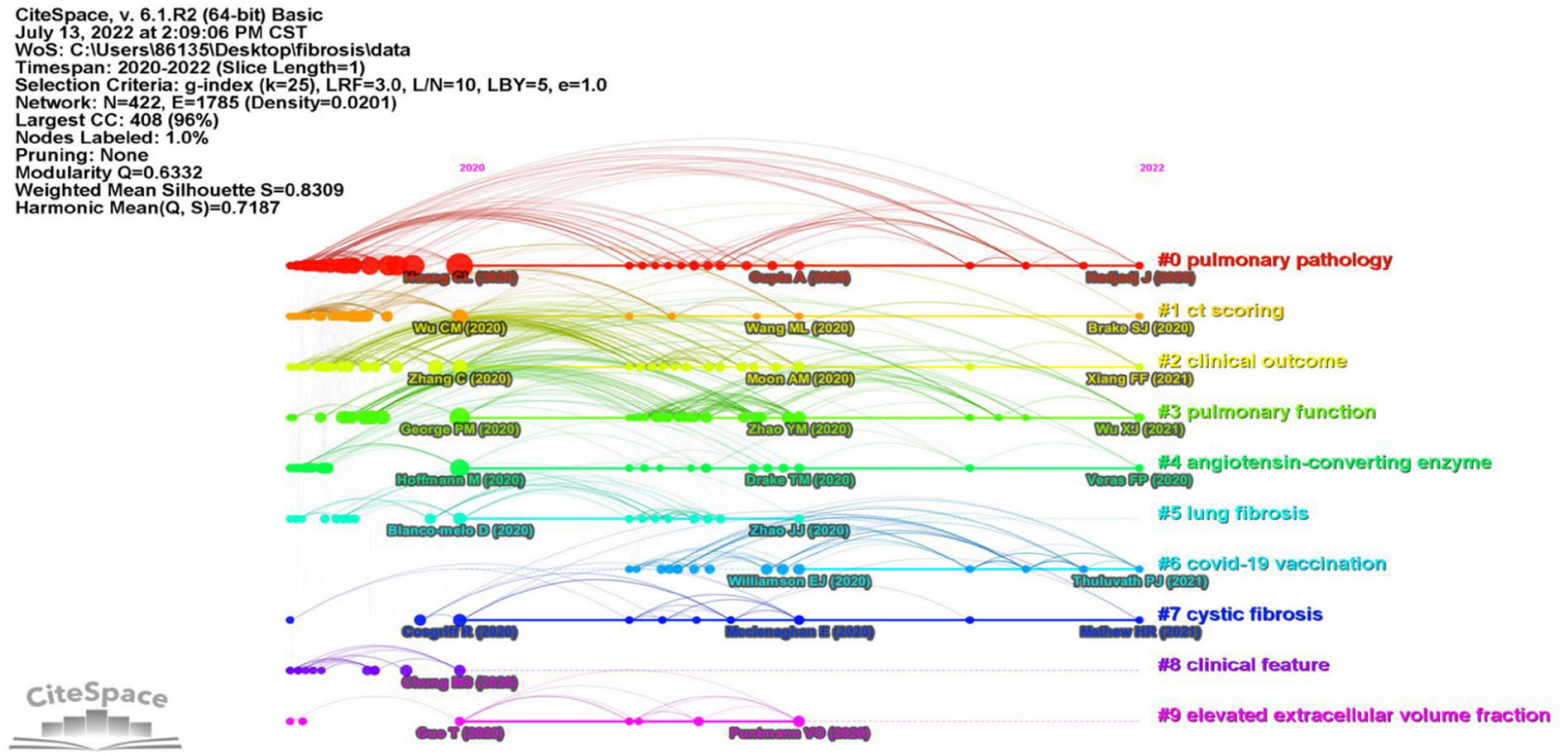
References co-citation time view generated by CiteSpace. Colors indicated different reference clusters. Labels of clusters and main references were automatically generated by CiteSpace.

References co-citation analysis resulted in a map composed of 422 bubbles representing cited references. The total modularity Q-value was 0.6332, indicating moderate integrated clustering. The mean silhouette score was 0.8757 indicating high internal consistency. Ten major clusters are distributed in the time view of reference co-citation (Figure 3), together with labels automatically generated with keywords. The labels of each cluster were “pulmonary pathology” (cluster 0), “ct scoring” (cluster 1), “clinical outcomes” (cluster 2), “pulmonary function” (cluster 3), “angiotensin-converting enzyme” (cluster 4), “lung fibrosis” (cluster 5), “COVID-19 vaccination” (cluster 6), “cystic fibrosis” (cluster 7), “clinical feature” (cluster 8), and “elevated extracellular volume fraction” (cluster 9), respectively. The size of the bubble indicated reference frequency. The axis showed the time span of clustered references. The time view represented the topic of co-cited reference changed from “pulmonary pathology” to “COVID-19 vaccination” (Figure 3).

In addition, Table 2 shows the top 10 most co-cited references on PCF, and most of them belonged to clusters 0. Among these, five references were clinical studies, two papers were *in vitro* studies, while the other three publications were case reports, reviews, and view points, respectively. The “Clinical features of patients infected with 2019 novel coronavirus in Wuhan, China” published in *Lancet* by Huang et al. from the Jin Yin-tan Hospital, Wuhan, China was the most co-cited reference (Frequency 170), reporting brief epidemiological and clinical profiles of patients with COVID-19.

**Table 2:** The ranking of top 10 most co-cited publications in the field of COVID-19-associated fibrosis.

**Rank**	**Author**	**Reference title**	**Source**	**Study type**	**Year**	**Frequency**	**Cluster ID**
1	Huang CL	Clinical features of patients infected with 2019 novel coronavirus in Wuhan, China.	Lancet	Prospective study	2020	170	0
2	Zhou F	Clinical course and risk factors for mortality of adult inpatients with COVID-19 in Wuhan, China: a retrospective cohort study	Lancet	Retrospective cohort study	2020	124	0
3	Guan W	Clinical Characteristics of Coronavirus Disease 2019 in China	New Engl J Med	Observational study	2020	108	0
4	Xu Z	Pathological findings of COVID-19 associated with acute respiratory distress syndrome	Lancet Resp Med	Case report	2020	100	0
5	George PM	Pulmonary fibrosis and COVID-19: the potential role for antifibrotic therapy	Lancet Resp Med	Review	2020	94	3
6	Hoffmann M	SARS-CoV-2 Cell Entry Depends on ACE2 and TMPRSS2 and Is Blocked by a Clinically Proven Protease Inhibitor	Cell	In vitro study	2020	89	4
7	Zhu N	A Novel Coronavirus from Patients with Pneumonia in China, 2019	New Engl J Med	In vitro study	2020	82	0
8	Wu ZY	Characteristics of and Important Lessons from the Coronavirus Disease 2019 (COVID-19) Outbreak in China: Summary of a Report of 72 314 Cases from the Chinese Center for Disease Control and Prevention	JAMA	View point	2020	66	0
9	Wang DW	Clinical Characteristics of 138 Hospitalized Patients With 2019 Novel Coronavirus–Infected Pneumonia in Wuhan, China	JAMA	Retrospective case series	2020	65	0
10	Ackermann M	Pulmonary Vascular Endothelialitis, Thrombosis, and Angiogenesis in Covid-19	New Engl J Med	Comparative Study	2020	58	0

### Country-wise analysis

**Figure 4:**
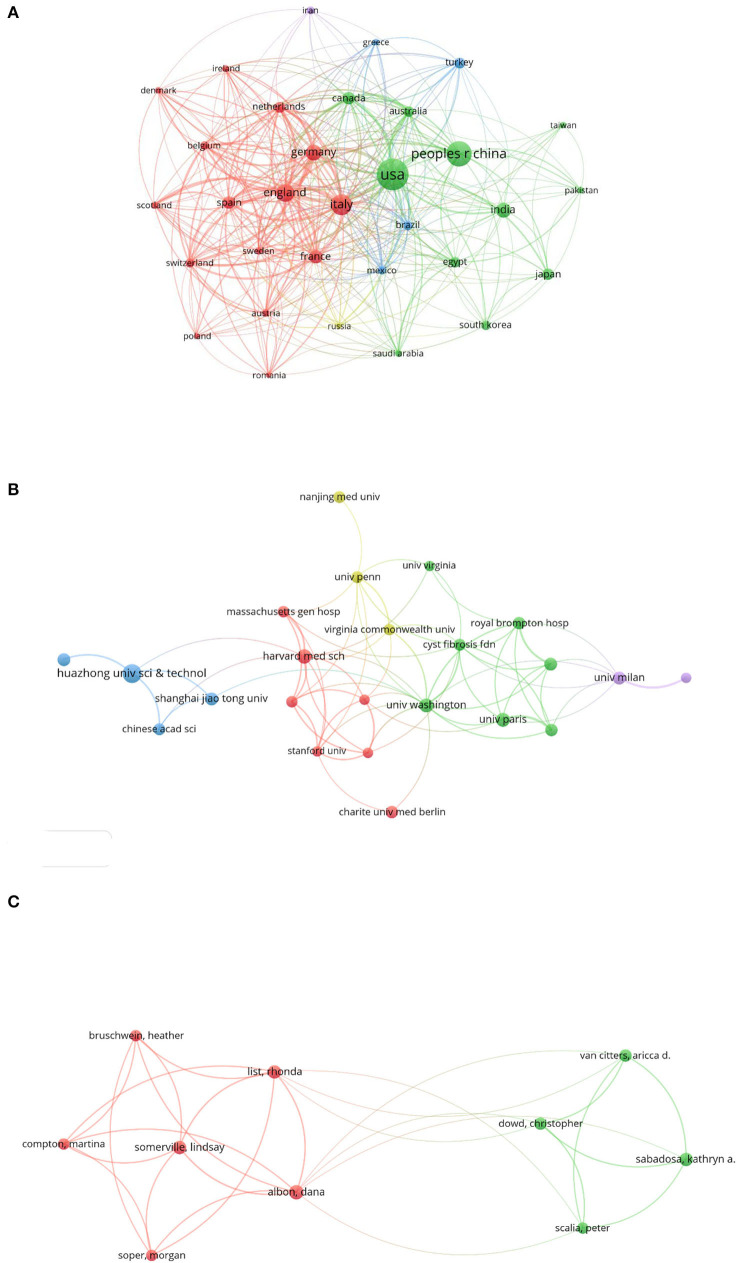
Collaborations in the field of COVID-19 associated fibrosis. **(A)** Country/region-wise co-authorship. **(B)** Institution-wise co-authorship. **(C)** Individualize co-authorship. Different colors indicated distinguished clusters. The size of the bubble indicated publication counts. The thickness of the line indicated linkage strength.

From the retrieved data, the most active countries in terms of the amounts of publications were demonstrated. The USA stood out first with 293 publications and 3,857 citations, followed by China, Italy, England, and Germany with 190, 128, 96, and 78 publications, respectively (Table 3). The country-wise collaborations are shown in Figure 4A and Supplementary Figure 3. Co-authorship between the USA (total link strength 227) and China were strongest (link strength 30 and total link strength 81), followed by England (link strength 25 and total link strength 209) and Germany (link strength 19 and total link strength 161), which were main partners of USA.

**Table 3:** Top 10 prolific countries/regions.

**Rank**	**Country/region**	**Publications**	**Citation counts**	***H*-index**
1	USA	293	3,857	30
2	Peoples R China	190	3,754	30
3	Italy	128	1,762	19
4	England	96	1,662	18
5	Germany	78	409	10
6	India	67	1,049	16
7	France	57	714	12
8	Spain	49	802	15
9	Canada	46	562	10
10	Japan	39	243	8

### Institution-wise analysis

A total of 2,383 institutions published papers in the emerging area of post-COVID-19 fibrosis. Among these, 24 institutions published more than 10 publications. The list of the top 10 productive is shown in Table 4. Udice French Research University won the first rank publishing 48 papers, followed by Institut National de la Sante et de la Recherche Medicale Inserm, Harvard University, Assistance Publique Hopitaux Paris Aphp, and Egyptian Knowledge Bank Ekb publishing 37, 34, 33, and 31 papers, respectively. However, the Imperial College London has the highest citation of 892. The highest H-index was 13, belonging to Harvard University. In terms of co-authorship, the University of Washington topped the total link strength of 26. It was closely tied with Cystic Fibrosis Foundation with the highest link strength of 4 (Figure 4B).

**Table 4:** Top 10 productive institutions.

**Rank**	**Institution**	**Publications**	**Citation counts**	***H*-index**
1	Udice French Research Universities	48	691	12
2	Institut National de la Sante et de la Recherche Medicale Inserm	37	633	11
3	Harvard University	34	724	13
4	Assistance Publique Hopitaux Paris Aphp	33	405	10
5	Egyptian Knowledge Bank Ekb	31	527	8
6	Huazhong University of Science Technology	31	694	11
7	Universite de Paris	28	396	10
8	Imperial College London	23	892	8
9	Harvard Medical School	22	573	10
10	Sorbonne Universite	20	329	9

### Author-wise analysis

To present the activity and impact of authors, the ranking of the top 10 prolific authors sorted by the number of publications is shown in Table 5 accompanied by citations and H-index. The most productive author was Dana Albon with eight publications. After this, Rhonda List, Lindsay Somerville, Kathryn A. Sabadosa, and Christopher Dowd ranked from 2 to 5, with 7, 7, 7, and 6 publications, respectively. To assess the impact of authors, H-index was exploited. Aricca D. van Citters topped the ranking with an H-index of 21. Individualized co-authorship was analyzed using VOSviewer. A total of 7,973 authors were extracted. Among these, 10 authors published more than five papers. The author-wise collaboration is shown in Figure 4C. Dana Albon, from the University of Virginia, USA, interested in telehealth for cystic fibrosis, observed the strongest link strength of 32. The main collaborators were Lindsay Somerville (link strength with Dana Albon, 7; total link strength, 28) and Rhonda List (link strength with Dana Albon, 6; total link strength, 30) from the University of Virginia, USA.

**Table 5:** The ranking of top 10 prolific authors.

**Rank**	**Author**	**Publications**	**Citation counts**	***H*-index**
1	Albon, Dana	8	64	5
2	List, Rhonda	7	63	3
3	Somerville, Lindsay	7	61	4
4	Sabadosa, Kathryn A.	7	24	15
5	Dowd, Christopher	6	20	7
6	Van Citters, Aricca D.	6	18	21
7	Bruschwein, Heather	5	61	4
8	Compton, Martina	5	61	3
9	Soper, Morgan	5	59	4
10	Scalia, Peter	5	14	9

### Journal-wise analysis

The ranking of the top 10 journals that published most papers on post-COVID-19 fibrosis is compiled in Table 6. *Journal of Cystic Fibrosis* topped the list with a total publication of 24, followed by the *Frontiers in Medicine, Plos One, Frontiers in Immunology*, and *Liver International* with 15, 15, 14, and 10 publications, respectively. Source H-index was enrolled to identify the impact of journals. *Journal of Cystic Fibrosis* stood out with the highest H-index of 9, followed by *Frontiers in Immunology, Journal of Hepatology, Frontiers in Medicine*, and *International Journal of Infectious Disease* with H-index of 7, 7, 6, and 6, respectively (Supplementary Figure 4).

**Table 6:** The ranking of top 10 journals that published most papers on post-COVID-19 fibrosis.

**Rank**	**Journal**	**Publications**	**Citation counts**	***H*-index**
1	Journal of Cystic Fibrosis	24	254	9
2	Frontiers in Medicine	15	100	6
3	Plos One	15	127	6
4	Frontiers in Immunology	14	234	7
5	Liver International	10	246	6
6	Medical Hypotheses	9	134	6
7	Journal of Clinical Medicine	9	65	4
8	Frontiers in Pharmacology	9	49	3
9	Scientific Reports	9	36	3
10	Hepatology	8	134	5

## Discussion

Accumulating publications documented pulmonary sequelae in COVID-19 survivors (6). It was reported that significant radiographic, spirometry, and laboratory abnormalities were observed weeks after recovery of COVID-19 (14). Therefore, a holistic understanding of PCF is paramount. In this study, an in-depth bibliometric analysis in the field of PCF has been performed to reveal the intrinsic structure, development, landmark, and trends of research regarding PCF.

### Overall structure of research in PCF

Currently, national, organizational, and personal level analysis of research in PCF were conducted. The results revealed that contributions mainly come from North America and Europe. Researchers from Asia including China, India, and Japan also played a crucial role in the field of PCF. Accordingly, PCF attracted worldwide attention as COVID-19 retained a global health threat to date. The *H*-index of China appeared equal to the USA, and a majority of most co-cited references come from China. This situation might imply an emerging contribution from China and growing attention to these researchers.

Nevertheless, the co-authorship total link strength of the USA, England, Italy, and Germany were 227, 209, 166, and 161, respectively, implying collaboration between North America and Europe was strong. In the contrast, China observed a moderate total link strength of 81, while other countries from Asia presented mild-link strength, indicating insufficient international collaboration in these countries. It was hypothesized that localized investigation was conducted due to the isolation of Asian countries during the global epidemic of COVID-19.

According to the ranking of co-citation references, clinical studies especially observational studies were prominent, targeting epidemiological, pathological, and radiographic profiles of fibrotic injury due to COVID-19. However, the *in vitro* and *in vivo* studies were limited resulting inadequate understanding of the etiology and mechanisms of this special disease, which is mandatory for the identification of efficacy agents against post-COVID-19 pulmonary fibrosis. Therefore, it is a necessity to pay attention to *in vitro* and *in vivo* studies for further holistic knowledge of COVID-19-induced fibrosis. In the near future, global cooperation should be facilitated for prospective, large sample size, multi-country investigations in an attempt to find out promising therapeutic therapies to restore the sequela of COVID-19.

### Dynamic evolution of hotspots and trends in the field of PCF

#### Study areas of considerable concern

To identify hot study areas, visualizations of high-frequency keywords in PCF were generated by various bibliometric tools. The epidemiology, physiopathology, diagnosis, and therapy of PCF were extensively studied.

Clinical evidence revealed etiological contributors to PCF, including acute respiratory distress syndrome (ARDS) following COVID-19 pneumonia and relevant sepsis (12), inflammation irregulating (25), viral pneumonia (38, 39), hyperoxia (40), diffuse thromboembolism (41), and other potential issues (42).

Antifibrotic therapies against fibrosis due to COVID-19 were also reported. Antifibrotic agents involving pirfenidone and nintedanib (43, 44), immune inhibitors (45), mesenchymal stem cell (MSC) therapy (46, 47), lung transplantation (27, 48), prolonged oxygen support, and rehabilitation exercise (49) might exert beneficial effects in preventing pulmonary fibrosis following COVID-19.

#### Research hotspots in the early stage of the COVID-19 epidemic

In the early phase after COVID-19 spread, long-term sequelae of the pandemic were just beginning to be concerned. Attention mainly focused on the pathogenic properties of the SARS-CoV-2 virus (22). Consistently, dynamic analysis of keywords and co-cited references indicated that pulmonary pathology of PCF involving “inflammation,” “cytokine storm,” “acute respiratory syndrome,” and “enzyme” were extensively studied in such an early stage.

##### The role of cytokine storm in the fibrosis process

The immunopathogenic phase of COVID-19, also known as the cytokine storm occurred ~10 days after the onset of infection (50, 51). Then, a sudden deterioration developed leading to a potentially fatal outcomes (52). However, the potential contributors to virus-provoked inflammatory responses were not well understood (51). McDonald et al. summarized the dual effect of cytokine release. On one hand, cytokines facilitated wound healing, on the other hand, dysregulation of inflammatory cytokines might be damaging (39). In COVID-19 patients, excessive release of cytokine, including IL1-β, IL-2, IL-6, IL-7, IL-8, IP10, MCP1, MIP1A, and tumor necrosis factor-α (TNF-α), exacerbated COVID-19 manifestation (2, 52, 53). Proinflammatory cytokine accumulation promoted dendritic cells exaggeration, lymphocyte stimulation, macrophage activation, neutrophil recruitment, immune-cells migration, exosome-mediated crosstalk induction, and ultimate tissue damage including fibrosis (18, 19). In addition, another cytokine transforming growth factor beta (TGF-β) was paramount in the fibrosis initiation and remodeling process (20). Emerging studies suggested that the TGF-β level was correlated with pulmonary fibrosis and interstitial pulmonary fibrosis (6, 54). Nonetheless, cytokine-leading neutrophilic infiltrate was reported to generate reactive oxygen species (ROS), and subsequently activate fibroblasts that ultimately deposit collagen and other extracellular matrix molecules to restore the virus-induced lung injury (55, 56). However, the aberrant repair process would aggravate tissue function due to complex contributors especially pulmonary fibrosis (39).

##### ARDS: the largest contributor to covid-19 related fibrosis

ARDS was common secondary to COVID-19, ~31.5% of patients developed ARDS during hospitalization (57). In the course of ARDS, pulmonary fibrosis occurred as early as <1 week (4%), with the incidence elevating rapidly beyond the third week (61%) (13). The pathogenesis underlying is complex (6). Prolonged mechanical ventilation (MV) induced thoracic injury with an exaggerated reparative response may be the largest contributor (14, 15). It was documented that fibroproliferative signaling cascades were initiated by MV stretching of the alveolar, followed by apoptosis and necrosis of type II alveolar epithelial cells (58). MV distortion and alveolar collapse-induced hypoxia further worsen this insult and aggravated pulmonary fibrosis (59). Furthermore, current evidence revealed that aberrant immunologic processes contributed to the fibroproliferation in COVID-related ARDS (39). Wendisch et al. described that SARS-CoV-2 triggered CD163-expressing monocyte-derived macrophages, which were similar to profibrotic macrophages acquired from idiopathic pulmonary fibrosis samples (60). In addition, high oxygen fraction during prolonged MV might trigger an oxygen-free radical cascade activating pro-fibrotic responses (41, 61). Other conserved pathogenesis underlying fibrotic response due to post-COVID-19 ARDS was also demonstrated, including epithelial injury (16), endothelial injury (17), cellular senescence (62), and deposition of extracellular matrix (63).

#### Recent emerging hotspots and future direction of research on PCF

Currently, COVID-19 was still ravaging the world, and the consequences of long COVID were of boosting concerns and realizations. According to results of thematic evolution and co-cited references analysis, “quality-of-life,” “post-COVID,” and “COVID-19 vaccination” received recent and future attention.

##### Quality of life of patients with PCF

Pulmonary fibrosis might lead to persistent symptoms, gradual loss of lung function, and long-term disability (22). Besides, people with pulmonary fibrosis might have increased financial burden, less income, and deteriorated quality of life. In patients with COVID-19, quality of life was evaluated using the EuroQol five-dimension five-level (EQ-5D-5L) questionnaire (24, 64). It is documented that hospitalized patients presented more fatigue and dyspnea, abnormal chest imaging manifestation, poor pulmonary diffusion capacity, depression and anxiety, and impaired quality of life at 6-month follow-up (24). Hence, PCF should be prevented and treated to provide a comfortable long-term recovery (65).

##### Covid-19 vaccination

To date, there is no proven effective antifibrotic therapy against PCF (38). Accordingly, the need for prophylactic vaccination with safety and efficacy is paramount to mitigate the severity of the disease (41, 66). The commercially available vaccine included messenger RNA (mRNA) vaccine encoding the spike protein (67), adenovirus-based vaccine (68), and inactivated COVID-19 vaccination (69). Laboratory and clinical investigations were still accelerated to identify a new vaccine against SARS-CoV-2 virus variants (22). The effectiveness of two doses of mRNA vaccine against SARS-CoV-2 was 90–92% (70), while the effectiveness of the inactivated vaccine was more than 95% (69). As the vaccination effectively protected persons from being infected, long-term COVID-19 involving PCF was diminished (71).

## Limitations

Nevertheless, there were some limitations in this current study. First, only the WOS core database was searched, rather than Scopus, PubMed, and other commonly used database. There might be some relevant literature inevitably missed. Nevertheless, data from the different databases were tough to combine together in bibliometric tools. In addition, the citation-related analysis could not be conducted using PubMed data. Second, the hotspots were evaluated based on the highly frequent keywords or theme words, rather than those presented in milestone research, implying potential bias of hotspots. Third, the quality of papers was not taken into account, and the quality of outcomes was hard to grade consequently.

## Conclusion

With the emerging spread of COVID-19, long-term sequelae involving pulmonary fibrosis become a major health threat. According to the best of our knowledge, this is the first bibliometric analysis evaluating PCF. Outcomes gained from this study assisted professionals to better realize PCF and would guide future practices.

Epidemiology, pathogenesis, and therapeutics were study hotspots in the early phase of PCF research. As the spread of the COVID-19 pandemic and progress in this filed, recent attention shifted to the quality of life of patients and post-COVID comorbidities. Nevertheless, COVID-19 relevant infection and vaccination were speculated to be research trends with current and future interest.

However, the mechanism underlying PCF remained speculative. Appropriate supports and treatments for PCF also needed to be optimized in the future. Therefore, international cooperation as well as in-depth laboratory experiments were encouraged to promote further explorations in the field of PCF.

## Data availability statement

The original contributions presented in the study are included in the article/Supplementary material, further inquiries can be directed to the corresponding author/s.

## Author contributions

Conception, design, collection, and assembly of data: HZ and YZ. Administrative support: Z-YH and Q-YX. Provision of study materials: Q-YX and S-PX. Data analysis and interpretation: S-YM, RT, and J-HF. All authors contributed to the manuscript writing and final approval of manuscript.

## Funding

The authors gratefully acknowledge the funding from the National Natural Science Foundation of China (NSFC, No. 81970059), the Natural Science Research Project of Shanghai (20ZR1433000), and the Shanghai Rising Stars of Medical Talents Youth Development Program—Youth Medical Talents: Clinical Pharmacist Program [SHWSRS (2021)_099].

## Conflict of interest

The authors declare that the research was conducted in the absence of any commercial or financial relationships that could be construed as a potential conflict of interest.

## Publisher's note

All claims expressed in this article are solely those of the authors and do not necessarily represent those of their affiliated organizations, or those of the publisher, the editors and the reviewers. Any product that may be evaluated in this article, or claim that may be made by its manufacturer, is not guaranteed or endorsed by the publisher.
