# Supplementary material for: Scars of COVID-19: A bibliometric analysis of post-COVID-19 fibrosis
Source: Front Public Health. 2022 Sep 20;10:967829. doi: 10.3389/fpubh.2022.967829 (PMC9530282; doi:10.3389/fpubh.2022.967829)

Supplementary Figure 1. Thematic map. A two-dimensional thematic map was generated by biblioshiny. X-axis represented relevance centrality indicating the importance of a keyword; Y-axis showed the development density revealing the progress potential of a keyword. Notably, one cluster, consisting of 64 keywords involving “expression”, “inflammation”, “receptor”, “pulmonary-fibrosis” and “activation”, was positioned in the upper right quadrant. Accordingly, these keywords were well-developed and important themes.

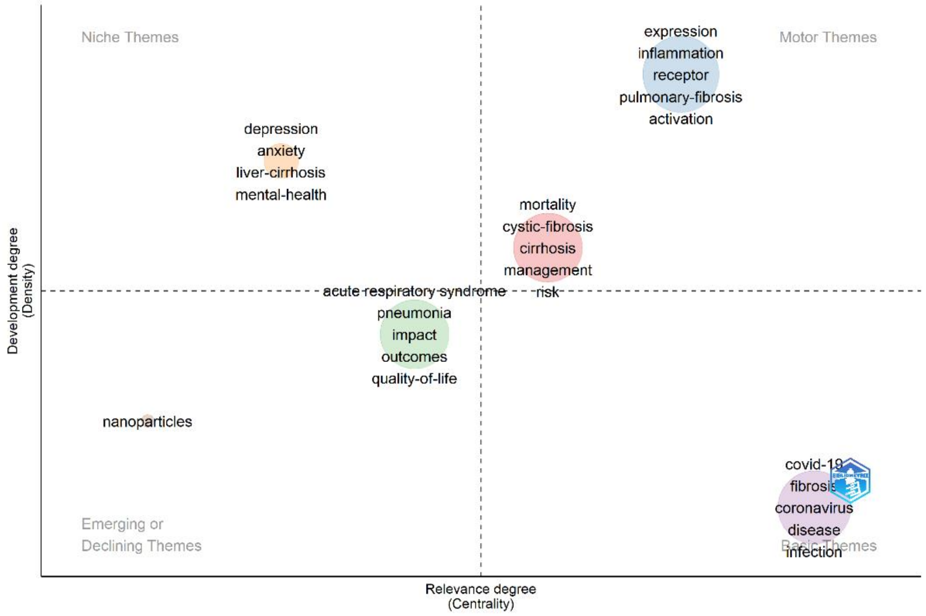

Supplementary Figure 2. Trends of study topics. Trend topics map was generated by biblioshiny. The circle size indicated term frequency. The line indicated the time span of term appearance. It showed the change of study topics during 2020-2022. The topics were "pneumonia", "wuhan", "outbreak" at the beginning of COVID-19 epidemic. Previously, the topics shifted to "covid-19", "fibrosis" and "expression" in 2021. Lately, "society", "epidemiology" and "database" turned to be new hot topics.

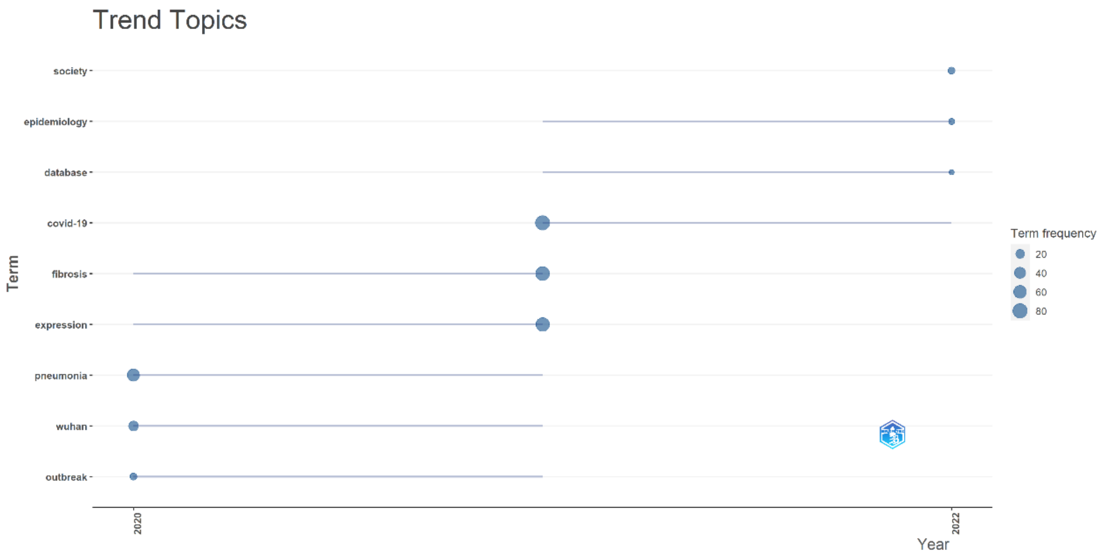

Supplementary Figure 3. Country/region wise publications and collaborations. The colors of the countries indicated the number of documents, that is, the darker blue implied more publications. The red lines revealed the linkage strength between countries, the thickness of which demonstrated the frequency of co-authorship between two countries or regions. The USA, China, Italy, England, and Germany ranked top 5 productive countries. The co-authorship link between USA and China were strongest, followed by China and Italy, which were main partners of USA.

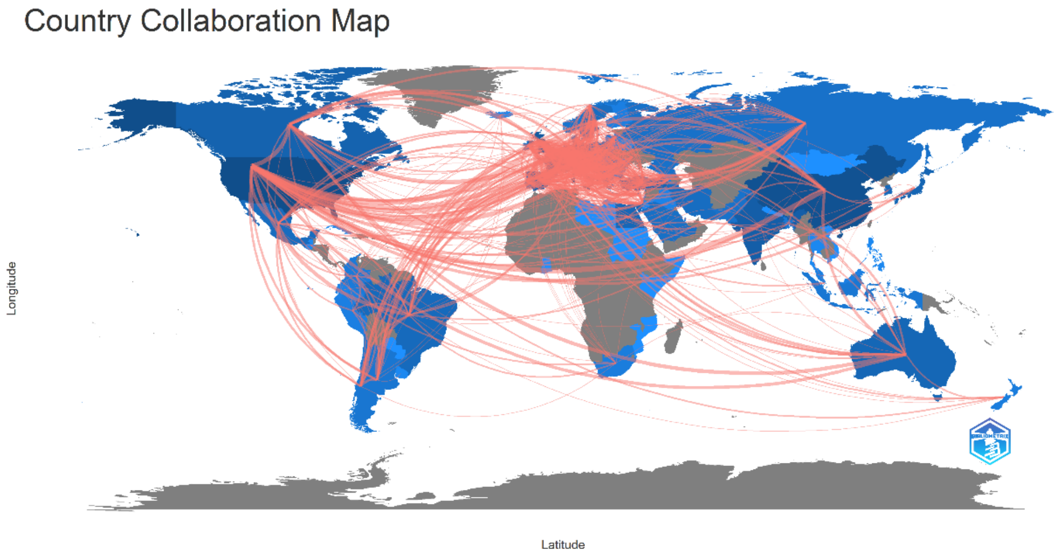

Supplementary Figure 4. The ranking of top 10 impacted journals. The X-axis indicated value of *H*-index. The Y-axis indicated journals. The circle size indicated value of *H*-index for particular items as well. The Journal of Cystic Fbrosis showed the largest *H*-index indicating high activity and influence.

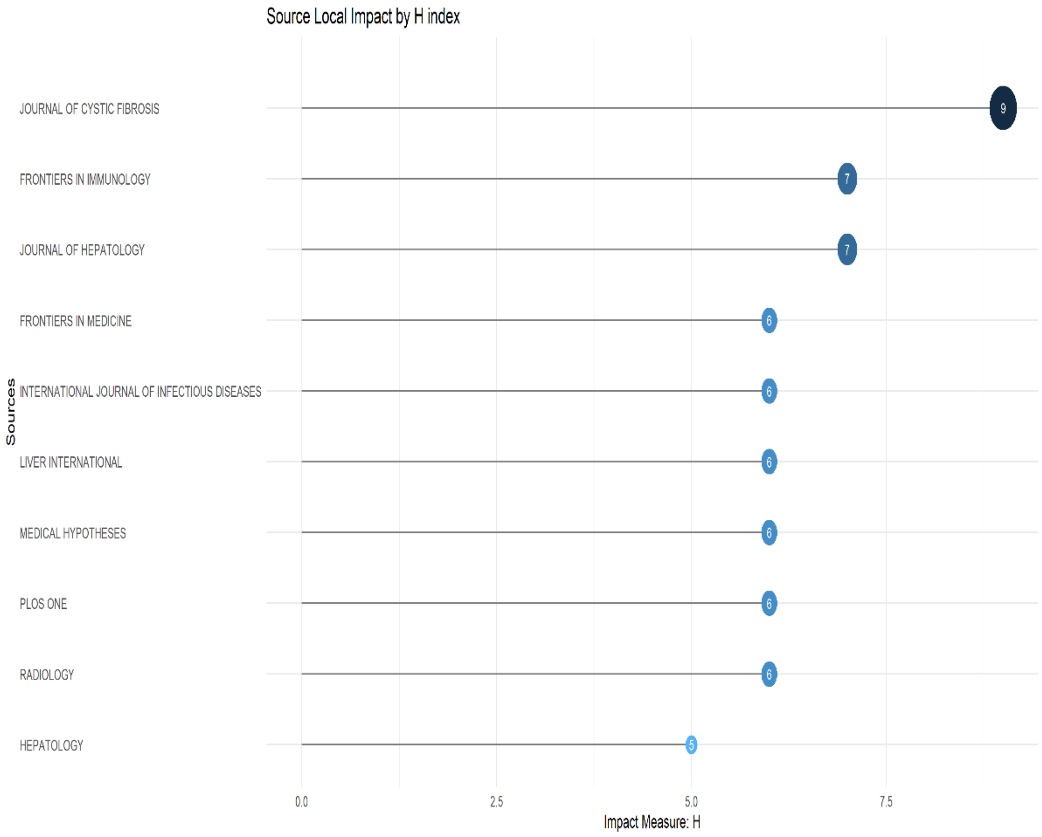

Supplement: Supplementary file 1 [file Presentation_1.pdf]
